# Let's talk about death: data collection for verbal autopsies in a demographic and health surveillance site in Malaysia

**DOI:** 10.3402/gha.v8.28219

**Published:** 2015-07-01

**Authors:** Pascale A. Allotey, Daniel D. Reidpath, Natalie C. Evans, Nirmala Devarajan, Kanason Rajagobal, Ruhaida Bachok, Kridaraan Komahan, The SEACO Team

**Affiliations:** 1 South East Asia Community Observatory (SEACO), Monash University, Segamat, Malaysia; 2 Global Public Health, School of Medicine and Health Sciences, Monash University, Malaysia; 3 Amsterdam Institute for Social Science Research (AISSR), University of Amsterdam, Amsterdam, the Netherlands

**Keywords:** verbal autopsy, cause of death, SEACO, health and demographic surveillance, beliefs, customs, bereavement

## Abstract

**Background:**

Verbal autopsies have gained considerable ground as an acceptable alternative to medically determined cause of death. Unlike with clinical or more administrative settings for data collection, verbal autopsies require significant involvement of families and communities, which introduces important social and cultural considerations. However, there is very little clear guidance about the methodological issues in data collection. The objectives of this case study were: to explore the range of bereavement rituals within the multi-ethnic, multi-faith population of the district; to investigate the preparedness of communities to talk about death; to describe the verbal autopsy process; to assess the effects of collecting verbal autopsy data on data collectors; and to determine the most accurate sources of information about deaths in the community.

**Methods:**

A case study approach was used, using focus group discussions, indepth interviews and field notes. Thematic analyses were undertaken using NVivo.

**Results:**

Consideration of cultural bereavement practices is importance to acceptance and response rates to verbal autopsies. They are also important to the timing of verbal autopsy interviews. Well trained data collectors, regardless of health qualifications are able to collect good quality data, but debriefing is important to their health and well being. This article contributes to guidance on the data collection procedures for verbal autopsies within community settings.

In most countries, death registration is a legal requirement. It is important for the purposes of monitoring where deaths occur, the age and sex distribution of who dies, and critically, the causes of death (1). The optimal standard is for highly accurate civil registration systems that provide up-to-date medically certified causes of death (2). When death occurs in a medical facility, cause of death is based on a clinical diagnosis. Where necessary, an autopsy or postmortem is performed (3). Where death occurs outside a health facility or within a home, there is an expectation that a medical practitioner would be available to examine the deceased, take a history, and certify a cause of death.

However, a significant number of deaths occur where there is no access to qualified medical staff (4). It is a particular challenge in low- and middle-income countries, where the infrastructure for registration is poorly developed, coupled with the shortage of human resources for health. Furthermore, there are many countries and cultures where the performance of autopsies is restricted by strongly held cultural and religious beliefs. The alternatives in these settings include the use of law enforcement authorities or community leaders such as teachers or headmen to provide a cause of death based on broad categories, such as ‘old age’ and ‘sudden death’ (5).

Over the last 50 years, therefore, resources have been put into developing efficient strategies to improve civil and particularly, cause of death registration (1, 2). Verbal autopsies, developed from more informal methods of lay reporting of health, have evolved into a systematic and standardised, indirect method to determine the biomedical cause of death on the basis of medical histories and signs and symptoms prior to death (6). The details are obtained from the deceased's carers (4, 7). Verbal autopsies have gained considerable ground as an acceptable alternative and have been used predominantly across health and demographic surveillance sites in the INDEPTH Network (8, 9). International standards and tools have been adapted and the intrinsic validity of aspects of the process has been ascertained (10). Verbal autopsies are now increasingly promoted by the World Health Organization to national ministries of health as data that need to be collected for determination of cause specific mortality (2, 11, 12). Verbal autopsies have also been used to determine social factors that contribute to mortality (13, 14) and to explore health systems related information (15).

The focus in the literature on improving and standardising verbal autopsies has, however, largely been on the analysis of the data (2, 12, 16–20), with very little clear guidance about the methodological issues in data collection. Unlike with clinical or more administrative settings for data collection, verbal autopsies require significant involvement of families and communities, which introduces important social and cultural considerations (21, 22). Guidance from the 2007 WHO manual is restricted to a recommendation that the appropriate person to interview is the primary caregiver with possible support from other family members. It also states that verbal autopsy interviews need to occur as soon as possible after the event, taking into account any culturally prescribed mourning period (11, p. 6). This guidance highlights the potential problems of recall bias but does not elaborate on the implications for support of families or the critical role of death rituals (23, 24).

Accessible publications that describe verbal autopsies are thin on detail about data collection. Some mention the duration of training of the data collectors (25), and others highlight the qualifications of data collectors (26). The most recent WHO manual provides a bit more detail about the training and competence of the interviewer but little else in terms of the data collection process (27, p. 17). There is significant variation in the time lapse between death and interview from 1 week (28) up to 13 months following the death (29); guidance from the WHO suggests as soon as possible and appropriate after the event but over 12 months should be interpreted with caution (27). Data sources on how to identify and compile deaths in the absence of access to registries are also inconsistently described.

A number of recent publications have raised concerns about the cultural and ethical considerations in talking to close family and carers about the deceased during bereavement (30, 31). Other concerns raised include the diagnostic accuracy of verbal autopsies for informing relatives about the cause of death, particularly if the determined cause of death has implications for insurance pay-outs or could be stigmatising for the family (30). The concerns suggest a greater need to understand the implications of collecting information on death from families and community to enhance their involvement in the process. The need for verbal autopsies, by definition, highlights the variation in contexts of obtaining the data and the challenges of standardising the methods for data collection. However, when promoting the method as an alternative to improving the cause of death data, it is important that during roll out, researchers have a clear understanding of the processes and their effects, on the response rates, the quality of the data, and on the well-being of those involved.

In the process of establishing the verbal autopsy protocols for the South East Asia Community Observatory (SEACO), a health and demographic surveillance site in Malaysia, we undertook a pilot study to field test the WHO verbal autopsy instrument selected for use in the site (27). As a part of the pilot test, a case study was designed to involve the community in developing the standard operating procedures for collecting verbal autopsy data. The objectives of the pilot study were: to explore the range of bereavement rituals within the multi-ethnic, multi-faith population of the district; to investigate the preparedness of communities to talk about death; to describe the verbal autopsy process; to assess the effects of collecting verbal autopsy data on data collectors; and to determine the most accurate sources of information about deaths in the community. Ultimately, we also aim at contributing to guidance on the data collection procedures for verbal autopsies within community settings.

## Methodology

### Research setting

SEACO is a demographic and health surveillance site in the district of Segamat, in the southern state of Johor, Malaysia. Established in late 2011, SEACO covers a population of approximately 40,000 (85% of population) from about 10,400 households in rural, semi-rural, and plantation areas in five sub-districts. The ethnic mix of the population reflects the national proportions of people of Malay (60%), Chinese (23%), and Indian (7%) descent (32).

Although Malaysia has a well-established civil registration system, about half of the deaths are not medically certified because they occur outside a health facility (5). Malays, as Muslims, require burials to occur as soon as possible after death, usually within 24 h. Routine autopsies are believed to desecrate the body and are not acceptable. Autopsies can only be authorised under a criminal procedures code if the police determine circumstances to be suspicious. As the predominant ethnic group, postmortems in Malaysia, regardless of ethnicity, are, therefore, only carried if there is a reasonable requirement.

For Hindus, autopsies are perceived as disturbing the still aware soul (22); cremations and, less frequently, burials, ideally occur within 24 h (33). Autopsies are more acceptable to Malaysia's Buddhists, but only once the ‘mind’ has actually left the body. This occurs, depending on the school of Buddhism, between 8 h and 3 days after death (33). During this time, the body should be moved as little as possible.

Verbal autopsies are, therefore, an important option to establish the cause of death. It is important to note that the national government of Malaysia is also in the process of instituting verbal autopsies as part of the death registration process, and the Ministry of Health is currently also pilot testing a Malaysia-specific verbal autopsy protocol in a number of states (5).

### Methods

A case study approach was taken with a combination of qualitative methods including focus group discussions, unstructured in-depth interviews, and field notes. Eight focus groups were organised with key informants from each of the five SEACO community engagement committees (described in detail elsewhere – see (21)), and with leaders from the local Hindu Temples, the Mosques, and the Chinese Clan leaders. The purpose of the focus groups was to explore the acceptability of verbal autopsies, obtain information on the details of death and bereavement rituals, and the appropriate times and protocols that SEACO staff would need to observe to collect verbal autopsy data. In-depth interviews with key informants were used to further probe this information to explore questions that included specifics on the appropriate persons to be present at the interview and any particular considerations associated with the type of death, the age and sex of the deceased, and the deceased's position in the family. Informants were also asked about the importance to the community, of knowing specific causes of death.

#### Training

A team of four data collectors for the verbal autopsies was identified from existing SEACO data collection staff based on criteria identified by the community. It is important to emphasise that the endorsement of the community for us was an essential criterion given the primacy of community empowerment in the community engagement strategy (21). From the community's perspective, data collectors for verbal autopsy interviews needed to be mature and discrete, able to empathise with families and respect bereavement rituals, and be known to or recommended by someone in the community.

In addition to the community criteria, we selected staff who also had formal qualifications or experience in public support roles; a retired nurse, a retired medical assistant, a retired teacher, and an active community volunteer. Prior to selection onto the verbal autopsy team, data collectors had previously undergone a total of 5 days of formal training to collect data for the SEACO census and updates and further 2 days for the SEACO health screening data collection round. ‘Classroom’ training for verbal autopsy data collection was conducted over a period of 2 days. This was followed by a day of supervised home-based verbal autopsy data collection to assess the ability of the data collectors to engage with families and administer the instrument. In addition, a debrief session was conducted after each data collector had done three interviews on their own to share the experience and discuss and improve the general approach to interviewing. The duration for the verbal autopsy training therefore built on their prior knowledge and experience of SEACO data collection procedures and ethos, and the verbal autopsy training could therefore focus predominantly on the reasons for and content of the instrument, management of distress amongst family members, and strategies for self-management of distress. The findings from the focus groups and interviews about bereavement were incorporated into the training as well. The training also reviewed their ability to work through the new technical additions to the Open Data Kit (ODK) form on the tablet (see below), particularly the skip logic functions based on the different protocols for different age groups and the recording and photography functions (11). The data collectors were supported by SEACO field supervisors and research and operations staff.

#### Tools

The WHO 2012 verbal autopsy instrument (27) was revised in consultation with researchers from the Malaysian Ministry of Health to take account of local conditions. These required, for instance, the inclusion of questions relating to dengue infections and removal of questions relating to conditions like malaria, which is not an endemic disease of significance in peninsular Malaysia. The choice of the instrument was to facilitate both local and international comparisons given standardisation, and to enable both physician and software assessments in the first instance (27).

The verbal autopsy instrument was then programmed into XML format which is interpreted by ODK Collect. ODK Collect is an open-source mobile application that is used primarily to collect questionnaire data on the Android based mobile devices used in SEACO. The instrument was programmed and validated as per SEACO standard operating procedures. Further additions were made to obtain an image file of the official death certificate and a function to switch on the device's audio recorder for any open-ended questions.

#### Identifying deaths

The national vital registry is protected by privacy laws and it was therefore not possible to use this source of data to identify deaths in the SEACO area. SEACO has, therefore, developed a verbal autopsy register compiling multiple sources that included deaths identified from the SEACO HDSS update round (conducted once a year), information provided by community key informants such as heads of hamlets and members of the SEACO community engagement committees, regular reviews of notices posted by ‘agents’ (undertakers), and obituaries in local newspapers. As a result of the range of community engagement activities, the notification of deaths by key informants has included individuals who were not enrolled in the SEACO. We have nonetheless reached out to the families to pay respects and where there has been interest, included the families and collected verbal autopsy data. Community key informants are not paid by SEACO for notification of deaths in the community.

On the basis of data from the second update round, 56 deaths were recorded in the SEACO sub-districts over the period between June 2012 and September 2014. These deaths were verified against the other sources cited above and verbal autopsies were undertaken for 55 of these for the pilot. There was one refusal from a family who did not want to relive the events leading up to the death by talking about it.

The verbal autopsy interviews were audio-recorded in their entirety to identify consistent areas of difficulty, both for participants and for data collectors. Particular areas of focus included the participants’ understanding of questions and strategies employed by data collectors in dealing with difficult questions and circumstances.

The community key informants currently appear to be the most comprehensive source of current information, providing notification to SEACO within a week of a death. However, multiple sources will continue to be maintained for the next 12 months as an ongoing method of validation. The ongoing triangulation of data sources also provides a mechanism to continue to improve quality of census update data on the SEACO database.

Ethics approval for the verbal autopsies (MUHREC Approval – CF14/317 – 2014000111) was obtained from the Monash University Human Research Ethics Committee as an add-on to the SEACO HDSS approval (MUHREC Approval CF11/3663 – 2011001930).

## Analysis

Focus group discussions, in-depth interviews, and verbal autopsy recordings were transcribed, translated in full, and analysed using a thematic approach. After a thorough reading of all transcripts, a coding scheme was developed and cross-cutting themes identified. Transcripts were managed and coded using NVivo qualitative data analysis software.

## Results

### Acceptability of verbal autopsies

Focus group discussions with members of the SEACO community engagement committees demonstrated a high level of community support for verbal autopsies. Members of the CECs saw the visits for interviews as an opportunity for them, as representatives, to enhance their visibility and show their support to members of their community. Several offered to accompany SEACO staff on initial visits to families to demonstrate to the community both their support of the SEACO initiative and their sympathy to bereaved families. Critically, the CECs confirmed the importance of acknowledging and adhering to the range of culture-specific bereavement practices across the various ethnic groups.

#### Pre-VA interview bereavement period

*Malay funerals*: As Moslems, burials for Malays take place within 24 h of death. The official mourning period reported by key informants is a maximum 3 days for a widower and up to 4 months and 10 days for a widow. However, the recommended time for VA interviews was at least 1 week of the death. The appropriateness of the time could also depend on the nature of the death and the age of the deceased and the discretion of the family or carers was the main consideration after a week's bereavement.

*Orang Asal*: The Indigenous Malaysians live in more remote rural communities of the district. A significant proportion of the Indigenous hamlets within the SEACO sub-districts have converted to Islam and as such are required to observe similar burial practices as the Malay community. Deaths occur predominantly in the home due to distance from and access to health facilities, and a preference, with illness, to remain with family. Deaths within the Orang Asal community are, therefore, certified predominantly by the police in order for families to obtain permits for the burial. The *Tok Batin* or chief of the village is a key player in supporting families in the Orang Asal community and has to be consulted before families are interviewed. The recommended time to approach families for verbal autopsies is at least a week after the death.

*Hindu*: Focus group discussions with elders from the Indian (predominantly Hindu) community highlighted the diversity of the specific rituals, which varied according to cast, family group, and particular tradition observed by the family. Furthermore, there was also a pluralism of practice across generations; even where parts of the family had converted to Christianity or Islam, there were beliefs and practices associated with death that anchored them to their ancestral roots. For instance, most families, regardless of religion, have a preference for deaths to occur within the home, surrounded by family. Speedy burial within 24–72 h was identified as important. Prayers to help the soul to cross over were observed for most families and had to be performed on the 8th and 16th days. For practising Hindu families, a further visit to the Temple is required on the 13th day. For these reasons, the earliest appropriate time for a VA visit to the family would be after 16 days. Regardless of the primary carer, people present at a verbal autopsy interview, where the circumstances surrounding the death were being discussed, would depend on whether the deceased was a child, married, or unmarried. There were also different considerations if a wife pre-deceased a husband or vice versa.

*Chinese community*: Discussions with Chinese key informants highlighted again the diversity of practice based on religion and cultural beliefs. For Buddhist families, the bodies are not touched by family until 10 h after death. This is followed by a period of chanting which can last for up to 4 days before burial can occur. Bodies are often laid out in the home for visitors to pay their respects. The duration of this depends on family wealth and prominence in the community. However, when the deceased is a child or unmarried, the body cannot be brought inside the house and therefore burial is likely to occur sooner. Key informants reported that there was also a perception that deaths clustered around the Dragon Festival month (*Pesta Perahu Naga* in the fifth lunar month) and the Hungry Ghosts month (Koh-tai seventh lunar month). These peak periods also affected how soon after the death burial can occur and therefore the preparedness of family to respond to verbal autopsies. Key informants suggested that requests for verbal autopsy interviews be avoided until at least 3 days after burial eventually occurred.

All ethnic groups required that the head of the household, regardless of whether he had been the primary carer of the deceased, be invited to the interview as a courtesy. It is worth noting that one of the challenges of waiting until after official mourning periods is that a carer may leave the rural family home to return to a life or employment in urban areas. A further issue was raised across all ethnic groups about the increasing number of elderly singleton households. Where a death occurred in the absence of any relatives, the closest neighbour provided the best option for verbal autopsy interview data in addition to any information on medical history that relatives would be able to provide.

The pre-interview process for SEACO is therefore as follows: when information is sent to the SEACO office about a death, a courtesy call is made to the family. Depending on the ethnic background of the family, an appropriate token is given from SEACO to convey condolences. Suggestions for tokens made by the community engagement committee ranged from garlands and wreaths to food hampers or a token cash contribution towards funeral arrangements. The value is modest and in line with what families would receive from neighbours coming to pay their respects. Ultimately, we sought advice from a family member or the *ketua kampung* (head of hamlet) about what would be most appropriate. The purpose of this visit is predominantly to offer condolences. This visit is also used as an opportunity to identify the carer of the deceased and to make an appointment for the interview for a follow up visit. Community engagement consultations noted that payment would not be necessary to households as a compensation for their time in providing data.

### Verbal autopsy process

Recordings of the entire verbal autopsy process revealed a number of implementation issues. For instance, it was sometimes difficult to ensure privacy during the interview at a time when there were often friends and relatives present to offer support. Data collectors, therefore, had to make alternative appointment times to ensure privacy which extended the period between the death and verbal autopsy interview. However, it was important that the verbal autopsy interviews were not perceived to take precedence over culturally appropriate family considerations.

There were also some problems in maintaining consistency in the language when data collectors needed to explain some of words in layman's terms. For example, a description of a saline drip was taken to mean dietary salt, and a question about a genetic disease was initially understood to be a question about blood loss. The two data collectors with health backgrounds tended to engage more with discussions about questions that were considered ‘technical’ although it was not clear that this provided any additional benefit to the veracity of the verbal autopsy data. It did, however, create opportunities to discuss broader health concerns within the family.

Data collectors also had to manage respondents’ occasional frustration at the length of the protocol, particularly with health questions seemingly unrelated to what they thought was a known cause of death. The more skilled data collectors stated clearly at the beginning of the verbal autopsy that all the questions were relevant even if they did not seem to be. However, it was also important to emphasise to the data collectors that the full instrument needed to be completed even if they sensed and empathised with the frustration of the respondent.

Questions about stigmatised conditions such as HIV proved difficult for the data collectors. They also found questions on alcohol consumption when the respondent was Muslim awkward because it implicitly questioned religious observance of a *haram* practice. Data collectors tried to avoid causing offense by distancing themselves personally from the question, and explicitly stating their discomfort about asking the question.

Most respondents found talking about the deaths emotionally stressful. It was not unusual for respondents to weep, particularly parents mourning (including adult) children. However, all were willing to continue and complete the interviews. There was a sharper recollection of and greater willingness to talk about events considered more emotive and personal, such as details of what happened on the day of the death. There were more difficulties recollecting dates in relation to medical histories and duration of illness, as well as in accurate information about the deceased's medical procedures and symptoms. Particular difficulties were noted with recall of details for deaths that had occurred more than 6 months prior to the interview. A key informant commented on the importance of the prior relationship of the data collectors to the community as a key factor in the good reception they got from the bereaved families.

#### Impact on data collectors

The data collectors selected for the verbal autopsy team all have prior experience collecting data for SEACO. They were, therefore, comfortable in the role, understood the research and its import, and were able to work well in supporting emotional distress and grief in the families. The data collectors with health backgrounds did, however, appear to cope better with the distress of the families. One of the data collectors reported that her empathy with the family was emotionally wearing and she found that the recording of field notes was like keeping a journal and was cathartic. However, all the data collectors were keen to maintain regular debrief sessions amongst themselves and with SEACO staff to share their experiences, talk about the challenges, and support each other.

Qualitatively exploring the interview process revealed that the administration of the verbal autopsy instrument was not neutral and objective. Rather, the information gained from their use was mutually constructed in a process of negotiation between data collectors and respondents. The flow of the interviews was dependent on the context in which the autopsies were conducted and the interpersonal skills of the data collectors. These findings emphasise the importance of careful recruitment of sensitive, local data collectors. They also highlight the need for training in ‘soft skills’ such as communication.

## Discussion and lessons learnt

This study attempts to provide some methodological detail about the processes involved in undertaking verbal autopsies. The experience for this pilot has informed the development of the standard operating procedures for verbal autopsies in SEACO.

In examining the implications of these findings to other settings, a number of issues arise. We achieved an almost 100% response rate for the families approached for verbal autopsy interviews (55 out of 56). The SEACO platform maintains and invests in an emergent model of partnership development with the community (21). Under this model, community relationships are prioritised in an effort to enhance participation in research and other interventions, in a spirit of long-term mutual benefit and ‘goodwill’. It would not be appropriate to test empirically the effectiveness of relationship building in achieving high response rates (34). However, the value of the SEACO approach to the community is evident through the media and other forums, which would support an assumption that it has contributed to the good response rates.

Existing literature does not provide any detail on response rates to verbal autopsy (5). Anecdotal reports from other settings that have attempted verbal autopsies outside a defined research setting suggest that response rates are low. The level of community engagement used in SEACO, however, may not be practicable where the implementation of verbal autopsies is for the purposes of civil registries or routine administrative data collection, as would be expected in a national scale up (2, 5). In the absence of a legal compulsion to respond to verbal autopsies (for example, as a condition of the issuance of a death certificate), one could anticipate that there would be challenges in achieving high response rates, and it is important that experiences in various settings is reported as a critical contribution to understanding of and refining verbal autopsy methods. A further consideration of response rates is whether the results of verbal autopsies are required for: epidemiological estimates of cause specific mortality, which may require no more than a sample; whether they are required by civil registries to support legal documentation or whether they are required for research or health and demographic surveillance.

The period between the death and verbal autopsy interview is also an issue that will require ongoing consideration. In some countries that are currently considering the national use of verbal autopsies, there is an expectation that verbal autopsies will produce the cause of death within a week in order to ensure integration with the civil registries and provide the evidence for legal death documentation (death certificates, insurance pay-outs, etc.). While this constrained period might address concerns about recall, significant pressure is placed on administrative authorities and this leaves no consideration for any cultural niceties. Data from our pilot highlighted some problems in recall within 6 months of the death but other studies suggest periods of up to 12 months (2, 13). The only empirical study undertaken to assess repeatability demonstrated that for a proportion of key responses required in the verbal autopsy protocol, reasonable recall can be demonstrated over a period of up to 2 years (10). There is, therefore, the need to strike a balance between the time required to observe cultural protocols and risk of recall bias. Recall bias has been an acceptable compromise for verbal autopsies to date because of the poor quality of data in most low income setting. However, with increasing recommendations for the method to be scaled up at the national level, careful consideration needs to be given to addressing this issue as a significant challenge. We would expect that in SEACO verbal autopsies will be conducted within 3 and no more than 6 months of a death.

Access to full audio recordings and qualitative analysis of the transcripts provides invaluable insights and will continue to be a routine part of data collection in verbal autopsies in SEACO. The full record of verbal autopsy interviews provides rich descriptions (to be reported elsewhere) that not only enable determination of cause specific mortality but can also contribute to our understanding of how communities understand chronic illness, terminal illness, death, and dying (35). These latter concepts and insights are critical to planning for community and home-based care of populations that are living longer and with increasing prevalence of chronic non-communicable diseases (36). The interviews also have the potential to reveal people's attitudes towards different types of deaths; informing debates about what constitutes a ‘good’ and a ‘bad’ death and the provision of end-of-life care in the region. Qualitative information obtained during verbal autopsies are also useful in determining attitudes, actions, and barriers to care (13).
